# How do women with social risk factors experience United Kingdom maternity care? A realist synthesis

**DOI:** 10.1111/birt.12446

**Published:** 2019-08-05

**Authors:** Hannah Rayment‐Jones, James Harris, Angela Harden, Zahra Khan, Jane Sandall

**Affiliations:** ^1^ Department of Women and Children's Health, School of Life Course Science, Faculty of Life Sciences and Medicine North Wing St. Thomas' Hospital, King's College London London UK; ^2^ Elizabeth Garrett Anderson Wing, University College Hospital London UK; ^3^ Institute for Health and Human Development, University of East London London UK; ^4^ Florence Nightingale Faculty of Nursing, Midwifery and Palliative Care King's College London London UK

**Keywords:** experiences of care, maternity services, socioeconomic status and ethnicity

## Abstract

**Background:**

Echoing international trends, the most recent United Kingdom reports of infant and maternal mortality found that pregnancies to women with social risk factors are over 50% more likely to end in stillbirth or neonatal death and carry an increased risk of premature birth and maternal death. The aim of this realist synthesis was to uncover the mechanisms that affect women's experiences of maternity care.

**Methods:**

Using realist methodology, 22 papers exploring how women with a wide range of social risk factors experience maternity care in the United Kingdom were included. The data extraction process identified contexts (C), mechanisms (M), and outcomes (0).

**Results:**

Three themes, Resources, Relationships, and Candidacy, overarched eight CMO configurations. Access to services, appropriate education, interpreters, practical support, and continuity of care were particularly relevant for women who are unfamiliar with the United Kingdom system and those living chaotic lives. For women with experience of trauma, or those who lack a sense of control, a trusting relationship with a health care professional was key to regaining trust. Many women who have social care involvement during their pregnancy perceive health care services as a system of surveillance rather than support, impacting on their engagement. This, as well as experiences of paternalistic care and discrimination, could be mitigated through the ability to develop trusting relationships.

**Conclusions:**

The findings provide underlying theory and practical guidance on how to develop safe services that aim to reduce inequalities in women's experiences and birth outcomes.

## 1 INTRODUCTION

Women living in areas with the highest levels of poverty in the United Kingdom are 50% more likely to experience a stillbirth or neonatal death.^1^, ^2^ These women experience increased rates of premature birth, low birthweight, cesarean, and maternal death.^3^, ^4^, ^5^, ^6^ As socioeconomic status decreases, women are more likely to report that they were not treated respectfully, that they were not spoken to in a way they could understand during their maternity care, and that their concerns are not listened to.^5^, ^8^ Health inequalities between socioeconomic groups are well documented^7^, ^8^ and have been a key priority in many international and United Kingdom initiatives, including the World Health Organization's (WHO) “Global strategy for women's and children's health”^9^ and the “Better Births” National Maternity Review.^10^

Lower socioeconomic status is often accompanied by other complex social factors associated with adverse outcomes^5^, ^11^, ^12^, ^13^, ^14^, ^15^ (Table 1). It is hypothesized that a lack of antenatal care and engagement with maternity services is directly linked to poor maternal and neonatal outcomes; therefore, policies are often focused on improving access to care.^9^, ^16^, ^17^, ^18^ A secondary analysis of the United Kingdom's National Maternity Survey^5^ showed that the most deprived women in the United Kingdom were 60% less likely to have received any antenatal care when compared to the least deprived women. Reviews of maternal and neonatal deaths^2^, ^3^, ^4^, ^14^ have found that women with social risk factors present real challenges for maternity services, with communication lapses between hospitals and the community health care setting.

**Table 1:** Social factors associated with increased risk divided into two groups 2, 3, 4, 5, 13, 15, 16, 19, 22, 25

Women who find services hard to access	Women needing multiagency services
Socially isolated	Safeguarding concerns
Poverty/deprivation/homelessness	Substance and/or alcohol abuse
Refugees/asylum seekers	Physical/emotional and/or learning disability
Non‐native language speakers	Female genital mutilation
Victims of abuse	HIV‐positive status
Sex workers	Perinatal mental health
Young mothers	
Single mothers	
Traveling community	

Marmots' review of the social determinants of health encourages the development of partnerships, with those affected by social inequities working with their health practitioners.^11^ Central to this approach is the development of a system that empowers women to have a real say in decisions that affect their lives, and that recognizes their fundamental human rights.^18^, ^19^ These values are echoed in the National Institute of Clinical Excellence (NICE) guidelines for women with complex social factors,^20^ which called for a reorganization of maternity services to improve antenatal care for this population and identified gaps in evidence with respect to effective service provision. Continuity of caregiver is a key government priority in an attempt to improve poor outcomes for women, with priority to be given to black and minority ethnic women alongside those living in the most deprived areas.^16^, ^17^ This is currently a far cry from the reality of a fragmented United Kingdom maternity system. A large, national United Kingdom survey^21^ reported 65% of women did not have a named midwife during pregnancy, and subgroup analysis of disadvantaged groups found inequalities in access to care, information, and interactions.^21^

Compared to women receiving standard care, a recently updated Cochrane review^24^ found that women who received continuity of care from a known midwife experienced significantly fewer preterm births, fetal losses, neonatal deaths, and clinical interventions and greater satisfaction. The review does not report on whether outcomes differed for socially disadvantaged women but recommended that future research should explore this population and the mechanisms underpinning the improved outcomes. Positive outcomes, including less clinical intervention, shorter hospital stays, fewer neonatal unit admissions, and increased liaison with multidisciplinary services for women with social factors, have been associated with continuity of care models in the United Kingdom.^25^, ^26^ There remains a paucity of evidence and professional agreement with respect to what models of care are effective in meeting specific population needs, and why some are more effective than others. Group antenatal care has also been identified as a possible way of reducing health inequalities for socially disadvantaged women, but the evidence to date is limited.^27^, ^28^ It is not known whether tailored models of care improve outcomes related to social deprivation, for example, the duration of breastfeeding, parent‐infant bonding, and childhood obesity. It is also not known how acceptable these models of care are for women with complex social factors, and whether they are seen as supportive, stigmatizing, or potentially isolating.

A systematic review^22^ found that the effectiveness of specific antenatal care programs to reduce infant mortality for socioeconomically disadvantaged women has not been rigorously evaluated. A further synthesis of women's views and literature focusing on the initiation of antenatal care by black and minority ethnic groups in the United Kingdom^23^ identified a range of barriers experienced by women including unfamiliarity with the system, inadequate interpretation services, a lack of cultural sensitivity, and impersonal care. The review suggested several interventions to overcome these barriers such as continuity of care, improved resources, and education but concluded that existing examples of specialist models of care should be fully evaluated and tested before they could be implemented into the wider NHS.

An analysis of the evidence presented in the Lancet series on midwifery^29^ recognized the importance of women's experience in improving clinical outcomes and indicated future research investment should address “right care‐ tailored to individuals, weighs benefits and harms, is person‐centred, works across the whole continuum of care, advances equity, and is informed by evidence, including cost‐effectiveness.” Therefore, this synthesis focused on how women with social risk factors experience maternity care in the United Kingdom, in order to advance theoretical understanding of the conditions required to increase the positive impact of care for this population. The aim was to uncover mechanisms that affect women's experiences of maternity care and develop program theories to be tested in a subsequent realist evaluation.

## 2 METHODS

Realist methodology attempts to understand what works, for whom, under what circumstances. It focuses on how people respond to interventions using contexts, mechanisms, and outcome configurations,^30^ for example, how women in a particular context respond to an aspect of their maternity care (the mechanism), and what is the outcome of this response. This was thought to be the most appropriate methodology for the review question posed as it not only recognizes the complexity of social risk factors and maternity services, but also allows the structured development of program theories to break these complex phenomena down into more manageable hypotheses to test what works in improving women's experiences of maternity care.

This synthesis was undertaken through regular collaboration with a patient panel consisting of recent maternity service users with social risk factors, and a panel of international experts in health inequalities and maternity care. Both panels advised on the review aims, search criteria, data extraction process, analysis, and identified gaps in the literature.

### 2.1 Literature search

**Figure 1:**
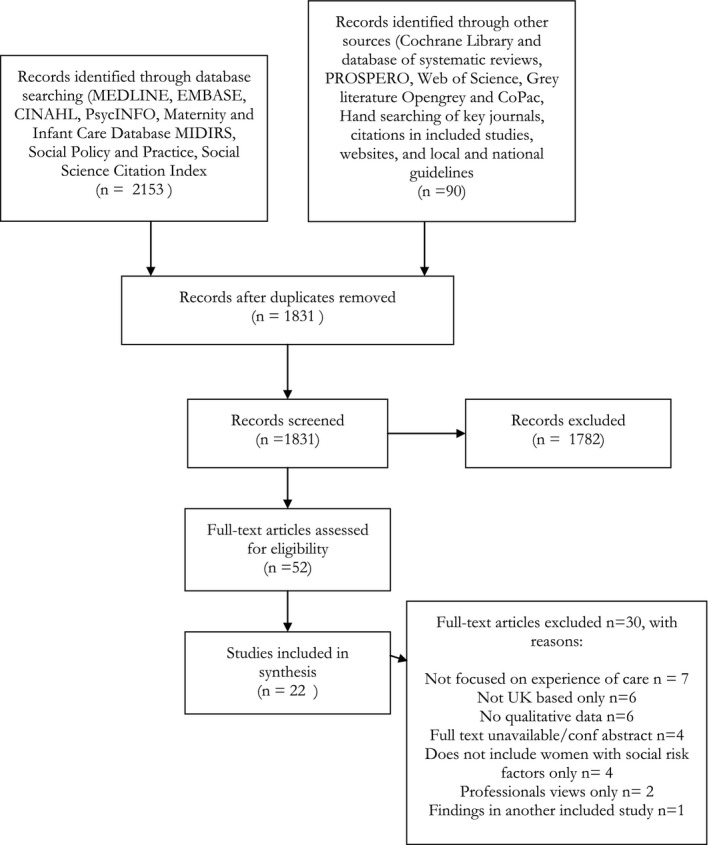
PRISMA Flow diagram

This realist‐informed, systematic synthesis of qualitative primary studies focused on the maternity care experiences of women with social risk factors using Pawson's^30^ 5 stages of a realist synthesis. Two independent researchers reviewed 1830 papers by title and abstract according to the search strategy and inclusion criteria (Table 2). Fifty‐two full‐text papers were reviewed and 22 papers included (Figure 1) (See Table S1 for an overview of included studies). Included studies were quality‐appraised using a validated checklist^53^ and generally assessed as high quality (Table 3). Although it was important to report on the quality of the studies, they were not weighted according to quality during the analysis as the purpose of this synthesis was to collate program theories and CMO configurations ready to test in a subsequent realist evaluation.

**Table 2:** Search strategy parameters and inclusion criteria in synthesis of how women with social risk factors experience United Kingdom maternity care

Facet	Definition	Search terms
Intervention	Included—United Kingdom‐based maternity care, including standard, routine care, and specialist models providing antenatal, intrapartum, and/or postnatal maternity care for women with social risk factors. Excluded—education programs, support groups, doula services, additional staff training, interventions/models of maternity care in any country other than the United Kingdom	Pregnan*, maternity, maternity care, maternity model, pregnancy care, model of care, maternal health service*, midwif*, obstetric*, healthcare, profession*, HCP, continuity, specialist, antenat*, intrapartum, postnatal, perinatal, team, intervention, birth
Participants/population	Women with low socioeconomic status and/or social risk factors identified in the working definitions	Social complex*, social Factor*, vulnerab*, socioeconomic, socioeconomic status, SES, depriv*, poverty, poor, disadvantag*, level of education, low education, low prestige, social class, disparit*, inequalit*, inequit*, discriminat*, impoverish*, low income, social* exclu*, social isolat*, homeless*, refuge*, immigra*, asylum*, non‐native language, language barrier*, minority ethnic*, ethnic*, black and minority ethnic, BME, sexual* abuse*, abuse*, domestic abuse*, domestic violence, intimate partner violence, IPV, physical abuse*, emotional abuse*, victim of abuse, sex worker*, adolescent*, young mother*, teenage*, single mother*, traveller*, travelling community, roma*, mental health, perinatal mental health, safeguard*, social care, social service*, child protection, substance abuse, drug abuse, addict*, alcohol*, alcohol abuse, disabil*, physical disabil*, learning disabil*, emotional disabil*, Female genital mutilation, FGM, Female circum*, HIV Positive status, HIV
Methodology	Included—qualitative literature or the qualitative data within mixed‐methods research Excluded—any literature published before 2010 to reflect the response to recommendations of the NICE^20^ maternity service guideline for women with complex social factors	Experien*, encounter, perception, view*, feel*, felt, remember*, recollect*, access*, engage*, communicat*, trust*, comfort*, uncomfort*

**Table 3:** Quality assessment of included papers in synthesis of how women with social risk factors experience United Kingdom maternity care^54^

References	Was there a clear statement of the aims of the research?	Is a qualitative methodology appropriate?	Was the research design appropriate to address the aims of the research?	Was the recruitment strategy appropriate to the aims of the research?	Was the data collected in a way that addressed the research issue?	Has the relationship between researcher and participants been considered?	Have ethical issues been taken into consideration?	Was the data analysis sufficiently rigorous?	Is there a clear statement of findings?	How valuable is the research?
Alshawish et al. 2013^31^	Y	Y	Y	Y	Y	Y	Y	Y	Y	Y
Baalam and Thomson 2018^32^	Y	Y	Y	Y	Y	N	Y	Y	Y	Y
Beake et al. 2013^33^	Y	Y	Y	Y	N	N	Y	Y	Y	Y
Bick et al. 2017^34^	Y	Y	Y	Y	Y	N	Y	Y	Y	Y
Binder et al. 2012^35^	N	Y	Y	N	Y	N	Y	Y	Y	Y
Bradbury‐Jones et al. 2015^36^	Y	Y	Y	Y	Y	N	Y	Y	Y	Y
Callaghan et al. 2011^37^	Y	Y	Y	Y	Y	N	Y	Y	Y	Y
Docherty et al. 2012^38^	Y	Y	Y	Y	Y	N	N	Y	Y	Y
Goodwin et al. 2018^39^	Y	Y	Y	Y	Y	N	Y	Y	Y	Y
Feldman et al. 2013^40^	Y	Y	Y	N	Y	N	N	N	N	Y
HESTIA 2018^41^	N	Y	N	N	Y	N	N	N	N	Y
Hatherall et al. 2016^42^	Y	Y	Y	Y	Y	N	Y	Y	Y	Y
Jomeen and Redshaw 2013^43^	Y	Y	Y	Y	Y	N/A	Y	Y	Y	Y
Lephard and Haith‐Cooper 2016^44^	Y	Y	Y	Y	Y	N	Y	Y	Y	Y
Malouf et al. 2017^45^	Y	Y	Y	Y	Y	N	Y	Y	Y	Y
McLeish and Redshaw 2018^46^	Y	Y	Y	Y	Y	N	Y	Y	Y	Y
Montgomery et al. 2015^47^	Y	Y	Y	Y	Y	N	Y	Y	Y	Y
Moxey et al. 2016^48^	Y	Y	Y	N	Y	Y	Y	Y	Y	Y
Phillimore 2016^49^	Y	Y	Y	Y	Y	N	Y	Y	Y	Y
Phillips and Thomas 2015^50^	Y	Y	Y	Y	Y	Y	Y	Y	Y	Y
Puthussery et al. 2010^51^	Y	Y	Y	Y	Y	N	Y	Y	Y	Y
Thomson et al. 2013^52^	Y	Y	Y	Y	Y	N	Y	Y	Y	Y

Abbreviations: N/A, Not applicable; N, No; Y, Yes.

### 2.2 Data extraction

A data extraction tool was devised and completed for each paper to identify explanatory contexts (C), mechanisms (M), and outcomes (0), and to develop program theories arising from these configurations. Program theories were constructed using “*if….., then…*” sentences. For example, “migrants who arrived in the country late in their pregnancy or had re‐located or been re‐dispersed from elsewhere in the UK (C), were unable to register with a GP in sufficient time to access maternity services before birth (O)” was converted into the following program theory: “*If* women who arrive in the country late in their pregnancy or have been re‐located or re‐dispersed from elsewhere in the UK are able to book maternity care directly with a midwife, *then* barriers to early access will be overcome and those who have difficulty registering with a GP will not be excluded.”

This process ensured transparency in converting findings into tangible, testable hypotheses or “program theory.” A total of 354 program theories were constructed from the findings of the 22 included studies. This collected the voices of 936 women with various social risk factors. Program theories were organized using data analysis software^53^ to uncover themes and develop middle‐range theories as recommended by Forster e al^55^ to increase transparency in decision making. This process enabled similar theories to be condensed, the extraction of theories specific to certain social risk factors, and the identification of conflicting theories. These conflicting theories give insight into what works in different contexts and for different populations.^56^ Once all papers had been classified according to the social risk factors included and the model of maternity care received and similar program theories condensed, 85 program theories remained. These final theories were grouped into the most commonly occurring themes and further refined into eight CMO configurations.

Middle‐range theories help conceptualize complex reality so that empirical testing of the more specific program theories becomes possible and generalizable.^57^, ^58^, ^59^ This conceptualization aided the development of the final CMO headings and has enabled a theoretically informed approach to the design of the subsequent realist evaluation, with the theories incorporated into the interview guides.

## 3 RESULTS

The full findings of this synthesis are detailed in 85 program theories (45 general theories and 40 that are specific to different social risk factors) and referenced to relevant included studies to demonstrate transparency (see Table S2). For the purpose of presenting a concise overview, the program theories were refined into eight overarching CMO configurations under three thematic headings (Table 4): System Resources, Relationships, and Candidacy. The CMO configurations are not ordered in relation to importance as all are thought to be important in impacting outcomes depending on the specific contexts identified. Quotes from women are included to add meaning and illustrate findings in the included studies.

**Table 4:** Context, mechanism, and outcome configurations. Results of realist synthesis of literature exploring how women with social risk factors experience United Kingdom maternity care

	Context	Mechanisms	Outcomes	Supportive quotations
CMO Configuration 1—access	Women who are unfamiliar with the NHS system, do not speak English and/or do not have a permanent United Kingdom address, asylum seekers, refugees, trafficked women, those experiencing domestic abuse	Written information (in a woman's preferred language) about how to access health services Direct access to maternity services rather than referral from a general practitioner (GP) The ability to access antenatal care without extensive documentation and without fear of disclosure to agencies or individuals who might put them at risk (eg, border agencies or embassies) Early access to maternity care (from conception/confirmation of pregnancy) Ability to rebook missed appointments with ease and without reproach	Earlier access to services, avoidance of denial of service, increased candidacy, increased autonomous choice through early access to safe abortion and family planning services	“*When I was 4‐5 months pregnant… I snuck out of the house and went to the local GP [family doctor] practice. When I arrived, they told me I needed a passport and proof of address. I explained that I didn't have this documentation and they turned me away*”^34^ “*They said to me, until we are sure that it's safe you see, to carry on with the pregnancy, then you can have a booking*”^42^
CMO Configuration 2—interpreter services	Women who do not speak English and those who have difficulties communicating (learning or physical disabilities)	Uncomplicated telephone access to interpreter services or online provision to register with services, arrange or reschedule appointments, organize travel to appointments, and to access advice from a health care professional Access to properly translated, language appropriate materials Choice of interpreter, for example, a female, an anonymous, or a trusted interpreter. Access to interpretation services throughout antenatal, intrapartum, and postnatal period, including emergency admissions	Earlier access to services, avoidance of denial of service, improved safety, flexibility, inequity in information received, increased confidence in help‐seeking and self‐disclosure	“*The problem we asked about an interpreter but unfortunately I didn't see her during my pregnancy 9 months*.”^31^ “*I asked them, ‘[Can] we cancel the meeting until we get an interpreter… I didn't understand you and you didn't understand me’. She said, ‘No, it's OK, we can go on—you understand English*’.”^44^
CMO Configuration 3—antenatal education	Women who may have limited education, unfamiliar with the system, language barriers, learning difficulties, caring responsibilities, no support, engage in “risky” behaviors	Culturally sensitive antenatal education (eg, child‐friendly settings and classes without the presence of men), with an opportunity for women to openly discuss cultural beliefs and advice received elsewhere Understandable, evidence‐based information that is well translated, about maintaining a healthy pregnancy, the impact of risky behaviors, routine procedures, and help‐seeking/support seeking	Increased candidacy, engagement with services, knowledge, choice, informed consent, help‐seeking, and lifestyle/behavior change	“*I never attended the antenatal class, because no one takes care of [my] other two kids. Where [can I leave] them?*”^31^ *Not enough information provided they give you leaflets and tell you some risks, but I would have liked to have talked to someone. It is different reading it than talking to someone and sometimes you don't understand the leaflets. So talking is better* 52
CMO Configuration 4—practical support	Women with a lack of resources/money/support, unfamiliarity with the United Kingdom culture and systems, frequent dispersal, socially isolated, learning disabilities, drug/alcohol abuse, undergoing child protection assessments	Provision of new skills/resources, for example, infant feeding support, provision of breast pumps, bottles and storage bags, reassurance, and motivation to abstain from illegal substances HCP's knowledge, time, and skill to coordinate and facilitate practical support to meet women's wider needs, for example, providing information about statutory procedures, contacting social workers, writing letters on women's behalf, coordinating and attending meetings with other statutory agencies (eg, social care, housing departments, home office) HCP's knowledge of maternity benefits and local support available to enable the provision of advice around practical matters such as housing, employment, education, and care of other children and family members	Women better prepared and supported for the challenges of parenthood and able to demonstrate their ability in parenting assessments, evidence of care and empathy from HCP's, increased agency, value in engaging with services, avoidance of further financial hardship, distress, and isolation. Development of a supportive network	“*[They] came to meetings when Social Services came to see us on the ward. They'd chat to us before and afterwards. They'd give us private rooms… to go and talk in if we needed to, away from the ward. They were fantastic emotionally, they were really supportive.*”^32^ “*I have asthma and I couldn't afford medication. I struggled when I was pregnant. I felt like I couldn't breathe and worried I'd harm my baby. My advocate helped me get the HC2 form so I could have my medication for free. I wish I'd known from the start.*”^41^
CMO Configuration 5—Continuity of care	Women living chaotic lives who struggle to access and engage with current, fragmented maternity services, social isolation, lack of resource, frequent dispersal, temporary accommodation, lack of support, complex social and/or medical history, disempowered, previous trauma or adverse experiences with services	Access to a known midwife or small team of midwives 24/7 by means of a phone call, text message, or free technology (freephone number, WhatsApp, Skype, etc) Continued supportive presence throughout pregnancy and the perinatal period, with a known midwife, GP, or other HCP who will coordinate communication across different trusts and services such as GP, gynecology, maternity services, social care, and mental health services HCP's work in a small geographical area where they are visible and become known by other members of the community, religious networks and other “gatekeepers,” local charities, food banks, befriending programs, and support services Flexible, needs‐led care, where the time and place of appointments is co‐planned (eg, at home, community, or a hospital setting, not at school times for single mothers, outside working hours for women working illegally)	Personalized, holistic care, increased engagement, trust, agency, candidacy, empowerment, sense of control, support, community integration, safety. Women are less likely to have to repeat their history and experience a variation of responses/advice, fragmentation/disassociation between services, and reduce stress/anxiety	“*Every time I saw the midwife during pregnancy and labour, I felt that I was just being processed, there was no opportunity to develop a relationship*.”^41^ “*Have one midwife—I think it would be much better for me. You understand… so I can… because the midwives there is different, and I don't know how to open to them. I can't be open up to a lot… every different people. When it's one person, then you can open up*.”^37^
CMO Configuration 6—relationship/ trust building	Women with previous and/or current experience of trauma, abuse, and discrimination, perceptions of previous manipulation and coercion by professionals, social isolation, lack of resources and support, limited education, unfamiliarity with systems and processes, complex social and/or medical history, disempowered, lack of sense of control, social care involvement/parenting assessments	Development of a trusting relationship with a known HCP through continuity, open discussion and story sharing, and the provision of meaningful, relevant information Provision of advocacy through known HCP attendance at meetings, and other forms of emotional support during interactions with social care Women are informed of their right to choice through education and provision of the evidence‐based information required to exercise that choice The perception of a health care professional to be respectful, understanding, kind, and helpful. Conflicting theory: It is more important that the whole service is perceived as safe, respectful, understanding, and kind, rather than one trusted HCP in a wider toxic environment	Meaningful interactions, self‐disclosure, increased perceptions of trust, empowerment, control, support, self‐confidence, shared decision making, knowledge of unfamiliar processes. Restore previously broken trust in systems/services and quash the belief that accessing care equates to relinquishing control and feeling violated. Avoidance of labeling women or making assumptions about their needs based on a perceived cultural background	“*I had built a relationship with her, I felt looked after and I had confidence in who was providing my care*”^52^ “*I would ask why was that and they were like, “Oh, it's our choice. It's our decision.” And just felt like we didn't have a say in in how…we could have our son…felt like we were invisible really…no need for us to even be there because they'd already made a decision*.”^45^
CMO Configuration 7—overcoming assumptions	Women who experience disadvantage, discrimination, stigma, and stereotyping based on their race, class, ability, age, and other sources of oppression	HCP's recognition of strengths and assets held by women and communities and respect for women's expertise of their own body, needs, and baby Recognition that women with social risk factors are more likely to experience paternalistic care, as passive recipients Women are encouraged to raise concerns in an easy and confidential manner and escalate those concerns if they are not satisfied with the response HCP's work within a community where they are immersed in local cultures and acknowledge the importance of culture and the influence of family members on women's experience of pregnancy	Women will not feel their cultural needs are being disregarded in favor of the Western medical model and inequities in access, engagement, the uptake of screening, and antenatal education will be reduced. Increased perception of being cared for on a personal level and involved in decision making. Avoidance of disempowerment, feelings of being pressurized, ignored, and excluded, long‐lasting psychological trauma, and increased control, bonding between a mother and her baby, improved self‐confidence, and potential adverse outcomes could be avoided	“*I were drip grey, my veins were closing up, and [the doctor] said, “Right, we'll break your waters now.” I said, “There's no way you can break my waters now, I need to go on a glucose drip, I'm really quite poorly, ‘and he said, ‘Oh, are you a doctor now?’ … And I said, ‘No I'm not a doctor, but I have lived with this condition since I were 15,’ and he actually looked at me and said, ‘What condition?’*”^46^ “*Sometimes there is quite a lot of jargon and when I go to my appointments you know when I'm being measured and stuff like that and they're checking for the foetal position and stuff they're not really telling back to me, I've got to come back and check my notes*.”^38^
CMO Configuration 8—surveillance	Women who fear judgment of health care professionals or perceive maternity services as a system of surveillance rather than support, for example, those with immigration issues who are worried that they can be tracked by authorities and their babies removed if they registered with services, trafficked women, young mothers, those with disabilities, women experiencing abuse, drug, and alcohol abuse, known to social care/undergoing parenting assessments	HCP's knowledge about reporting mechanisms for women with immigration issues, including processes of payment as a non‐United Kingdom resident, and ability to signpost women to confidential advice HCP's ability to explain the reasoning behind reporting safeguarding concerns, the process of assessment, and discussion of what “meaningful support” means to the woman Women's involvement in the process of reporting safeguarding concerns in an open manner that encourages them to identify their needs Processes are in place that protect the woman from being put at risk of harm, for example, women whose abusers or traffickers may control or observe access to services are given the opportunity to self‐disclose in safe environment and disclosures are followed up safely and sensitively	Increased access and engagement, self‐disclosure, trust, safety, development of meaningful support networks, improved long‐term outcomes for mother and child. Decreased intergenerational vulnerability, discrimination, disconnectedness, fear, and anxiety	“*I thought if you said something how you's exactly feeling, and if you was feeling a bit down that particular day, that they would use that against you*”^45^ “*It is safer not to ask for help, you'd better Google rather than ask midwives… I didn't want them thinking, ‘Oh, she can't do it’*”^46^

The Resource theme included (a) access to maternity services and (b) appropriate antenatal education, (c) interpreter services, (d) practical support, and (e) continuity of care, these were particularly relevant for women who are unfamiliar with the National Health Service (NHS) system and those living chaotic lives. For women with experience of trauma, abuse, and discrimination, or those who lack a sense of control, (e) the ability to build a relationship with a health care professional was key to regaining trust in the system and control over what happens to them and their baby. The “Candidacy” theme recognized that women with social risk factors are more likely to experience paternalistic care and highlighted the impact of (f) health care professionals' assumptions based on race, class, ability, age, and other sources of oppression. This might be overcome by placing services in local communities where health care professionals are immersed in local cultures and recognize the strengths and assets held by women and their communities. Lastly, many women with social risk factors perceive health care services as a system of (g) surveillance rather than support, impacting on engagement and meaningful support. This could be mitigated through the ability to develop trusting relationships, health care professionals' knowledge of safeguarding and reporting mechanisms, and processes put in place to ensure women's safety.

## 4 DISCUSSION

This synthesis systematically identified qualitative literature that focused on the experiences of maternity care in the United Kingdom for women with social risk factors and used realist methodology to uncover the contexts and mechanisms that led to positive or negative experiences. These contexts and mechanisms were coded and developed into CMO configurations, providing a set of program theories to test and compare women's experiences in future research and evaluation of services. The findings contribute to knowledge by providing detailed insight into how different social risk factors affect women's ability and willingness to access and engage with services. The realist methodology takes the findings of the 22 included papers deeper by unearthing potential mechanisms that may improve or worsen experiences.

Twenty of the 22 included studies reflected the views of standard maternity care in the United Kingdom reflecting the availability of specialist models of care for women with social risk factors. The included studies covered a range of social risk factors that were often multiple and overlapping. Black and minority ethnicity, and asylum seeker/refugee status were the risk factors most commonly focused on, and although the vast majority of the studies found that the participants were socially deprived, only four of the 22 papers used social deprivation in their inclusion criteria. By focusing on single social risk factors when designing research or services, the complexity of social deprivation and oppression may be overlooked and deficits within the system disregarded. For example, the growing body of literature on the “healthy migrant” phenomenon shows that many first‐generation immigrants often have better physical and mental health than the indigenous populations of many developed countries.^60^, ^61^ This suggests that it is not that a person is not native to a country that puts them at risk of health inequalities, but it is growing up in a place where that person might be perceived as different that has a greater bearing. This synthesis found that for black and minority ethnic women, asylum seekers, and refugees, it was the language barrier and unfamiliarity with the United Kingdom system that had the biggest impact on how they accessed, engaged, and experienced their maternity care. This leads us to the concept of intersectionality. Although intersectionality was not explicitly discussed in the included studies, it became a clear factor in how women experienced maternity care. Oppressive institutions of racism, sexism, ableism, classism, etc, are interconnected, impact on health inequalities,^62^ and cannot be separated when trying to understand why some women experience maternity care differently to others. One example of this is found in Bradbury‐Jones' study^36^ where the women felt that not only they were perceived as less able to make decisions because of their disability, but also this was compounded by health care professionals' judgments about the domestic abuse they had experienced.

Five of the eight CMO configurations related to system resources: access, interpreter services, education, practical support, and continuity of care. This closely reflects the findings of Hollowell et al's^23^ review of black and minority ethnic women's experiences of maternity care. A frequent finding in both papers was the importance of community‐based care, allowing women and midwives to integrate with the local community, and ease access to services for women who lack resources or are not able to travel far to hospital appointments.

The importance of relationships was so apparent in the program theories that it became a key middle‐range theory. There is a wealth of literature on the benefits of continuity of care on women's outcomes.^23^, ^24^, ^25^, ^26^ This synthesis found that for women whose trust has previously been broken, either through interactions with professionals, or previous trauma and abuse, the development of a trusting relationship with a health care professional results in increased confidence, safety, and empowerment. It also reduced women's perceptions of discrimination, manipulation, and coercion by people in power. Although “relationships” was found to be an occurring theme in this synthesis, the concept of trust was tied in closely to this. Women described the impact of trust in health care professionals and trust in the system as a whole. Literature on the theoretical perspectives of trust describes these two aspects, suggesting that trust in a person can act as a moderator/mediator when there is distrust in a system.^63^, ^64^ However, this protective factor is vulnerable to the trusted person not being there. A conflicting program theory identified that for some women, particularly those with social care involvement, it was more important that the whole service is perceived as safe, respectful, understanding, and kind, rather than one trusted professional in a wider toxic environment. The data from women who expressed this were linked to perceptions of surveillance, which may explain why the thought of one known health care professional might be perceived as intimidating, and building a relationship may be viewed as an invasion of privacy. It should be noted that the vast majority of included papers reflected standard maternity care and that those women who had experienced a form of continuity did not report negative perceptions of surveillance and valued the relationship they had with their health care practitioner/support person. Dismantling the belief that accessing health care services equates to relinquishing control may have long‐lasting consequences on women's social interactions, help‐seeking, and parenting. Conversely, if women with social risk factors, particularly those that contribute to disempowerment, experience paternalistic care through being denied choice and perceive health care professionals as lacking warmth, patronizing, arrogant, and stigmatizing, then they will remain disempowered and feel undervalued, and their low self‐confidence will increase.

Candidacy, defined as “the ways in which people's eligibility for medical attention and intervention is jointly negotiated between individuals and health services,”^65^ was the umbrella concept for two CMO configurations: “assumptions” and “surveillance.” The concept suggests that a woman's “candidacy” for maternity services is materially, culturally, and organizationally constructed. For example, it is well known that more deprived women access preventative health care services less than more affluent women,^5^, ^66^ and have higher use of emergency services.^67^ Candidacy is thought to be at play here, with factors such as help‐seeking in response to crisis symptoms rather than to prevent poor health, the normalization and acceptance of poor health, and fear of blame from health care professionals apparent across many of the included studies. Again, these factors were found in Hollowell et al's review,^23^ with barriers to initial access, lack of interpreter services, discrimination/disrespectful care, and health care professionals' lack of cultural knowledge affecting how women perceived their candidacy for services. The findings of this synthesis extend these findings further by proposing that if the value of accessing maternity services for the purpose of monitoring, prevention, and support is communicated across the communities in which women live, through community‐based services and relationship building, then women would not view the purpose of the service as simply the treatment of ill health, and access care earlier in pregnancy.

### 4.1 Strengths, limitations, and gaps in literature

Overall, the studies included in the synthesis were assessed to be of high‐quality and they reported on studies conducted with women with a range of different social risk factors. However, the number of studies reporting women's socioeconomic status was limited. Only two of the studies reported specialist models of care, with the remaining studies reflecting the experiences of standard maternity care. This meant that the development of program theories for what works in improving women's experiences was often drawn from negative experiences and inverted to a positive program theory. To test those theories, a full evaluation of how women experience specialist models of care is required.

A further limitation of the synthesis is the cutoff date of 2010 in the inclusion criteria (see Table 2), potentially restricting the depth of the findings. This criterion aimed to reflect the NICE^20^ guidance for women with social complex factors and to compare findings with previous systematic reviews of women's experiences of antenatal care.^22^, ^23^ With these limitations in mind, the findings of this synthesis add depth and detail in what works, for whom, in what circumstances, and how, to existing recommendations from the international wider literature.^5^, ^8^, ^9^, ^13^, ^18^, ^22^, ^23^

There were some themes that were expected to be reported but were not. These included the recognition of women's personal strengths and assets, and the impact of their community. This may be because the women interviewed felt these were not important, because the research approach did not explore these themes, or because they were not included in final published work. The assumption of deficit—that people are a burden on the state rather than a resource—with respect to low‐income people, asylum seekers, refugees, and migrants was sometimes apparent in the reported experiences of women but was not made explicit in the discussion sections of the studies. In addition to this, despite the growing body of evidence into the “healthy migrant effect,” the papers included in the synthesis did not explore inequities in health service use, experiences, and outcomes for second‐ or third‐generation descendants. Tudor Hart's^68^ “inverse care law”—the principle that those most in need of care are the least likely to receive it—was also evident in the findings of many included studies but not discussed. For example, do health care professionals “do more” for more affluent women? Do women with lower socioeconomic status have lower expectations of maternity services? Further research, using qualitative realist evaluation methodologies with all stakeholders, will help to answer these questions and test the program theories put forward in this synthesis.

### 4.2 Conclusions

The findings of this synthesis provide both an underlying theory and practical guidance on how to develop safe, person‐centered maternity services for women with social risk factors that encourage early access and meaningful engagement and reduce the discrimination and fear this group of women often experience. The synthesis contributes to knowledge by identifying how women with different social risk factors experience care in different ways, resulting in specific program theories tailored to more individualized need. The CMO configurations developed will be tested in a realist‐informed evaluation of two specialist models of care (one community based and one hospital based) within areas of significant health inequity in London, United Kingdom. The synthesis also highlights potentially significant gaps in the literature, such as the impact of discrimination on outcomes and experiences, potentially stigmatizing service provision, or the protective factors of community and family support. These knowledge gaps should be explored in future research and considered when planning services for this vulnerable population.

## References

[1] Seaton SE , Field DJ , Draper ES , et al. Socioeconomic inequalities in the rate of stillbirths by cause: a population‐based study. BMJ Open. 2012;2(3):e001100. 10.1136/bmjopen-2012-001100 PMC3383980 22735165

[2] Knight M , Tuffnell D , Kenyon S , Shakespeare J , Gray R , Kurinczuk J . Surveillance of maternal deaths in the UK 2011–13 and lessons learned to inform maternity care from the UK and Ireland confidential enquiries into maternal deaths and morbidity 2009–13 Updated 2015.

[3] Draper E , Kurinczuk J , Kenyon S . MBRRACE‐UK 2017 Perinatal Confidential Enquiry: Term, Singleton, Intrapartum Stillbirth and Intrapartum‐related Neonatal Death. Leicester: The Infant Mortality and Morbidity Studies, Department of Health Sciences, University of Leicester; 2017.

[4] Centre for Maternal and Child Enquiries (CMACE) . Saving mothers’ lives: reviewing maternal deaths to make motherhood safer: 2006–08. The eighth report on confidential enquiries into maternal deaths in the United Kingdom. BJOG. 2011;118(Suppl. 1):1‐203. 10.1111/j.1471-0528.2010.02847.x 21356004

[5] Lindquist A , Kurinczuk JJ , Redshaw M , Knight M . Experiences, utilisation and outcomes of maternity care in England among women from different socio‐economic groups: findings from the 2010 National Maternity Survey. BJOG. 2015;122(12):1610‐1617. 25227878 10.1111/1471-0528.13059

[6] Smith LK , Draper ES , Manktelow BN , Field DJ . Socioeconomic inequalities in survival and provision of neonatal care: population based study of very preterm infants. BMJ. 2009;339:b4702. 19952036 10.1136/bmj.b4702 PMC2786957

[7] Blumenshine P , Egerter S , Barclay CJ , Cubbin C , Braveman PA . Socioeconomic disparities in adverse birth outcomes: a systematic review. Am J Prev Med. 2010;39(3):263‐272. 20709259 10.1016/j.amepre.2010.05.012

[8] Redshaw M , Rowe R , Henderson J . Listening to Parents After Stillbirth or the Death of Their Baby After Birth. London: National Perinatal Epidemiology Unit; 2014.

[9] Ki‐Moon B . Global Strategy for Women's and Children's Health. New York: World Health Organisation, United Nations; 2010.

[10] NHS England . National Maternity Review. Better Births; Improving Outcomes of Maternity Services in England. London: NHS England; 2016.

[11] Marmot M , Friel S , Bell R , Houweling TA , Taylor S . Commission on Social Determinants of Health. Closing the gap in a generation: health equity through action on the social determinants of health. Lancet. 2008;372(9650):1661‐1669. 18994664 10.1016/S0140-6736(08)61690-6

[12] Khalifeh H , Hargreaves J , Howard LM , Birdthistle I . Intimate partner violence and socioeconomic deprivation in England: findings from a national cross‐sectional survey. Am J Public Health. 2013;103(3):462‐472. 22897532 10.2105/AJPH.2012.300723 PMC3673488

[13] World Health Organization . Social Determinants of Mental Health. Geneva: World Health Organization; 2014.

[14] Nair M , Knight M , Kurinczuk JJ . Risk factors and newborn outcomes associated with maternal deaths in the UK from 2009 to 2013: a national case–control study. BJOG. 2016;123(10):1654‐1662. 26969482 10.1111/1471-0528.13978 PMC5021205

[15] London Maternity Clinical Network (LMCN) . London Maternal Deaths. A 2015 Review. London, UK: NHS; 2016.

[16] Kennedy HP , Yoshida S , Costello A , et al. Asking different questions: research priorities to improve the quality of care for every woman, every child. Lancet Global Health. 2016;4(11):e777‐e779. 27663682 10.1016/S2214-109X(16)30183-8

[17] Department of Health . The Mandate: A Mandate from the Government to the NHS Commissioning Board: April 2013 to March 2015. London, UK: Department of Health; 2012.

[18] Ten Hoope Bender P , Homer C , Matthews Z , et al. The state of the world's midwifery: a universal pathway, a woman's right to health. 2014.

[19] George AS , Branchini C , Portela A . Do interventions that promote awareness of rights increase use of maternity care services? A systematic review. PLoS One. 2015;10(10):e0138116. 26444291 10.1371/journal.pone.0138116 PMC4596618

[20] National Institute for Health and Clinical Excellence . Pregnancy and Complex Social Factors: A Model for Service Provision for Pregnant Women with Complex Social Factors. London, UK: NICE; 2010. 31886994

[21] Care Quality Commission, (CQC) . Women's Experiences of Maternity Care in England: Key Findings from the 2015 NHS Trust Survey. London: CQC; 2015.

[22] Hollowell J , Oakley L , Kurinczuk JJ , Brocklehurst P , Gray R . The effectiveness of antenatal care programmes to reduce infant mortality and preterm birth in socially disadvantaged and vulnerable women in high‐income countries: a systematic review. BMC Pregnancy Childbirth. 2011;11(1):13. 21314944 10.1186/1471-2393-11-13 PMC3050773

[23] Hollowell J , Oakley L , Vigurs C , Barnett‐Page E , Kavanagh J , Oliver S . Increasing the early initiation of antenatal care by Black and Minority Ethnic women in the United Kingdom: a systematic review and mixed methods synthesis of women's views and the literature on intervention effectiveness. Social Science Research Unit, Institute of Education, University of London. 2012.

[24] Sandall J , Soltani H , Gates S , Shennan A , Devane D . Midwife‐led continuity models versus other models of care for childbearing women. Cochrane Database of Systematic Reviews. 2016(4). 10.1002/14651858.CD004667.pub5 PMC8663203 27121907

[25] Rayment‐Jones H , Murrells T , Sandall J . An investigation of the relationship between the caseload model of midwifery for socially disadvantaged women and childbirth outcomes using routine data–a retrospective, observational study. Midwifery. 2015;31(4):409‐417. 25661044 10.1016/j.midw.2015.01.003

[26] Homer CS , Leap N , Edwards N , Sandall J . Midwifery continuity of carer in an area of high socio‐economic disadvantage in London: a retrospective analysis of Albany Midwifery Practice outcomes using routine data (1997–2009). Midwifery. 2017;48:1‐10. 28284877 10.1016/j.midw.2017.02.009

[27] Catling CJ , Medley N , Foureur M , et al. Group versus conventional antenatal care for women. Cochrane Database of Systematic Reviews. 2015, (2). 10.1002/14651858.CD007622.pub3 PMC6465187 25922865

[28] Wiggins M , Sawtell M , Wiseman O , et al. Testing the effectiveness of REACH Pregnancy Circles group antenatal care: protocol for a randomised controlled pilot trial. Pilot Feasibil Stud. 2018;4(1):169. 10.1186/s40814-018-0361-x PMC6234800 30459959

[29] Kennedy HP , Cheyney M , Dahlen HG , et al. Asking different questions: a call to action for research to improve the quality of care for every woman, every child. Birth. 2018;45(3):222‐231. 29926965 10.1111/birt.12361

[30] Pawson R . Evidence‐based Policy: A Realist Perspective. London: Sage; 2006.

[31] Alshawish E , Marsden J , Yeowell G , Wibberley C . Investigating access to and use of maternity health‐care services in the UK by Palestinian women. Br J Midwifery. 2013;21(8):571‐577.

[32] Balaam M , Thomson G . Building capacity and wellbeing in vulnerable/marginalised mothers: a qualitative study. Women Birth. 2018;31(5):e341–e347. 29370993 10.1016/j.wombi.2017.12.010

[33] Beake S , Acosta L , Cooke P , Mccourt C . Caseload midwifery in a multi‐ethnic community: the women's experiences. Midwifery. 2013;29(8):996‐1002. 23415359 10.1016/j.midw.2013.01.003

[34] Bick D , Howard LM , Oram S , Zimmerman C . Maternity care for trafficked women: survivor experiences and clinicians' perspectives in the United Kingdom's National Health Service. PLoS One. 2017;12(11):E0187856. 29166394 10.1371/journal.pone.0187856 PMC5699814

[35] Binder P , Borné Y , Johnsdotter S , Essén B . Shared language is essential: communication in a multi‐ethnic obstetric care setting. J Health Commun. 2012;17(10):1171‐1186. 22703624 10.1080/10810730.2012.665421

[36] Bradbury‐Jones C , Breckenridge JP , Devaney J , Kroll T , Lazenbatt A , Taylor J . Disabled women's experiences of accessing and utilising maternity services when they are affected by domestic abuse: a critical incident technique study. BMC Pregnancy Childbirth. 2015;15(1):181. 26289166 10.1186/s12884-015-0616-y PMC4546038

[37] Callaghan M , Buller AM , Murray SF . Understanding ‘late bookers’ and their social circumstances. Br J Midwifery. 2011;19(1):7‐13.

[38] Docherty A , Bugge C , Watterson A . Engagement: an indicator of difference in the perceptions of antenatal care for pregnant women from diverse socioeconomic backgrounds. Health Expect. 2012;15(2):126‐138. 21615639 10.1111/j.1369-7625.2011.00684.x PMC5060614

[39] Goodwin L , Hunter B , Jones A . The midwife–woman relationship in a South Wales community: experiences of midwives and migrant Pakistani women in early pregnancy. Health Expect. 2018;21(1):347‐357. 28960699 10.1111/hex.12629 PMC5750740

[40] Feldman R . When maternity doesn't matter: dispersing pregnant women seeking asylum. Reprod Health Matters. 2013;21(42): 212‐217.

[41] HESTIA . Underground Lives: Pregnancy and Modern Slavery. London: HESTIA; 2018.

[42] Hatherall B , Morris J , Jamal F , et al. Timing of the initiation of antenatal care: an exploratory qualitative study of women and service providers in East London. Midwifery. 2016;36:1‐7. 27106937 10.1016/j.midw.2016.02.017 PMC4853798

[43] Jomeen J , Redshaw M . Ethnic minority women's experience of maternity services in England. Ethnicity Health. 2013;18(3):280‐296. 23039872 10.1080/13557858.2012.730608

[44] Lephard E , Haith‐Cooper M . Pregnant and seeking asylum: exploring women's experiences ‘from booking to baby’. Br J Midwifery. 2016;24(2):130‐136.

[45] Malouf R , Mcleish J , Ryan S , Gray R , Redshaw M . 'We both just wanted to be normal parents': a qualitative study of the experience of maternity care for women with learning disability. BMJ Open. 2017;7(3):e015526. 10.1136/bmjopen-2016-015526 PMC5372071 28341692

[46] Mcleish J , Redshaw M . Maternity experiences of mothers with multiple disadvantages in England: a qualitative study. Women Birth. 2019;32(2):178–184. 29910026 10.1016/j.wombi.2018.05.009 PMC7074001

[47] Montgomery E , Pope C , Rogers J . A feminist narrative study of the maternity care experiences of women who were sexually abused in childhood. Midwifery. 2015;31(1):54‐60. 24929272 10.1016/j.midw.2014.05.010

[48] Moxey JM , Jones LL . A qualitative study exploring how Somali women exposed to female genital mutilation experience and perceive antenatal and intrapartum care in England. BMJ Open. 2016;6(1):e009846. 10.1136/bmjopen-2015-009846 PMC4716221 26743705

[49] Phillimore J . Migrant maternity in an era of superdiversity: new migrants' access to, and experience of, antenatal care in the West Midlands, UK. Soc Sci Med. 2016;148:152‐159. 26705910 10.1016/j.socscimed.2015.11.030

[50] Phillips L , Thomas D . The first antenatal appointment: an exploratory study of the experiences of women with a diagnosis of mental illness. Midwifery. 2015;31(8):756‐764. 25975830 10.1016/j.midw.2015.04.004

[51] Puthussery S , Twamley K , Macfarlane A , Harding S , Baron M . ‘You need that loving tender care’: maternity care experiences and expectations of ethnic minority women born in the United Kingdom. J Health Serv Res Policy. 2010;15(3):156‐162. 20466754 10.1258/jhsrp.2009.009067

[52] Thomson G , Dykes F , Singh G , Cawley L , Dey P . A public health perspective of women's experiences of antenatal care: an exploration of insights from a community consultation. Midwifery. 2013;29(3):211‐216. 22341092 10.1016/j.midw.2012.01.002

[53] QSR International Pty Ltd . NVivo Qualitative Data Analysis Software. Doncaster: QSR International Pty Ltd Version 10, 2014.

[54] Critical Appraisal Skills Programme (CASP) . CASP Qualitative Research Checklist; 2017 http://docs.wixstatic.com/ugd/dded87_25658615020e427da194a325e7773d42.pdf. Accessed March 20, 2018.

[55] Forster N , Hodgson P , Dalkin S , Lhussier M , Carr S . Charting the impacts of Citizens Advice Bureau activities: strategies to orchestrate a realist analytical process. 2015.

[56] Pawson R . The Science of Evaluation: A Realist Manifesto. London: Sage; 2013.

[57] Merton RK . On sociological theories of the middle range In: Social Theory and Social Structure. New York: The Free Press; 1968:39.

[58] Jagosh J . Incorporating middle‐range theory in realist evaluation and synthesis. [Webinar]. June 2018. CARES Webinar Training Series.

[59] Shearn K , Allmark P , Piercy H , Hirst J . Building realist program theory for large complex and messy interventions. Int J Qual Meth. 2017;16(1):1609.

[60] Dhadda A , Greene G . ‘The healthy migrant effect’ for mental health in England: propensity‐score matched analysis using the EMPIRIC survey. Journal of Immigrant and Minority Health. 2018;20(4):799–808. 28389831 10.1007/s10903-017-0570-z PMC6061089

[61] Hayes L , White M , McNally RJ , Unwin N , Tran A , Bhopal R . Do cardiometabolic, behavioral and socioeconomic factors explain the ‘healthy migrant effect’ in the UK? Linked mortality follow‐up of South Asians compared with white Europeans in the Newcastle Heart Project. J Epidemiol Community Health. 2017;71:863‐869. 10.1136/jech-2017-209348 28743730

[62] Bowleg L . The problem with the phrase women and minorities: intersectionality—an important theoretical framework for public health. Am J Public Health. 2012;102(7):1267‐1273. 22594719 10.2105/AJPH.2012.300750 PMC3477987

[63] Gilson L . Trust and the development of health care as a social institution. Soc Sci Med. 2003;56(7):1453‐1468. 12614697 10.1016/s0277-9536(02)00142-9

[64] Meyer S , Ward P , Coveney J , Rogers W . Trust in the health system: an analysis and extension of the social theories of Giddens and Luhmann. Health Soc Rev. 2008;17(2):177‐186.

[65] Dixon‐Woods M , Cavers D , Agarwal S , et al. Conducting a critical interpretive synthesis of the literature on access to health care by vulnerable groups. BMC Med Res Methodol. 2006;6(1):35. 16872487 10.1186/1471-2288-6-35 PMC1559637

[66] Cresswell JA , Yu G , Hatherall B , et al. Predictors of the timing of initiation of antenatal care in an ethnically diverse urban cohort in the UK. BMC Pregnancy Childbirth. 2013;13(1):103. 23642084 10.1186/1471-2393-13-103 PMC3652742

[67] Scantlebury R , Rowlands G , Durbaba S , Schofield P , Sidhu K , Ashworth M . Socioeconomic deprivation and accident and emergency attendances: cross‐sectional analysis of general practices in England. Br J Gen Pract. 2015;65(639):e649‐e654. 26412841 10.3399/bjgp15X686893 PMC4582877

[68] Hart JT . The inverse care law. Lancet. 1971;297(7696):405‐412. 10.1016/s0140-6736(71)92410-x 4100731

