# Supplementary material for: How do women with social risk factors experience United Kingdom maternity care? A realist synthesis
Source: Birth. 2019 Aug 5;46(3):461–74. doi: 10.1111/birt.12446 (PMC6771833; doi:10.1111/birt.12446)
Supplement: Supplementary file 1 [file BIRT-46-461-s001.docx]

Supplementary table 1 (published online only): Characteristics of included papers in synthesis of how women with social risk factors experience UK maternity care

| First Author, date, reference | Aim and methodology | Participant, model of care |
| --- | --- | --- |
| Alshawish, 2013  (31) | To investigate access to and use of health services, particularly maternal and child health care, in the UK by Palestinian women.  In-depth interviews | 22 Palestinian women  Standard UK maternity care |
| Baalam 2018  (32) | To explore vulnerable/marginalised women’s views and experiences of receiving targeted support from a specialist midwifery service and/or a charity.  Mixed-methods involving analysis of routinely collected birth outcome data and in-depth interviews. | 11 women with complex social risk factors including: poor mental health, homelessness, substance use, social isolation, domestic abuse, having children in care, or being asylum seekers or refugees.  Additional targeted support from specialist midwifery services or a charity |
| Beake, 2013  (33) | To evaluate caseload midwifery in a relatively deprived and ethnically diverse inner-city area.  Semi-structured interviews | 24 women from diverse ethnic backgrounds  12 received caseload midwifery care  12 received standard maternity care |
| Bick, 2017  (34) | To explore health care needs, service use and challenges among women who became pregnant while in the trafficking situation in the UK and clinicians’ perspectives of maternity care for trafficked persons.  Cross-sectional survey and qualitative interviews | 28 pregnant trafficking survivors  Standard maternity care |
| Binder, 2012  (35) | To gain a deeper understanding of the multi-ethnic care setting and the roles that ethnicity and language play between immigrant women and their western obstetric care providers.  In-depth individual and focus group interviews using semi-structured, open-ended questions | 39 immigrant Somalian women, 11 Ghanaian women  Standard maternity care |
| Bradbury-Jones, 2015  (36) | To identify how disabled women who are affected by domestic abuse approach maternity care services, their expectations of services and whether they are able to get the type of care that they need and want.  A qualitative, Critical Incident Technique study | 5 women who had seen a health professional in relation to pregnancy; had experienced domestic abuse; and lived with a health condition or impairment (physical, mental health, sensory or intellectual)  Standard maternity care |
| Callaghan, 2011  (37) | To contribute to reducing the gap in the knowledge about ‘late booking’ for maternity care with a detailed exploration of such women’s own accounts and perspectives on their relationship with NHS pregnancy care.  Semi-structured, in-depth interviews. | 20 women representing differing social backgrounds and ethnic characteristics for instance, women from BME groups, particularly Black African and Bangladeshi women, teenagers and those living in socially deprived circumstances  Standard maternity care |
| Docherty, 2012  (38) | To determine whether pregnant women’s perception of antenatal provision differed in relation to their socioeconomic deprivation ranking.  Longitudinal, qualitative study with comparative antenatal case studies. | 12 women from ‘most deprived’ geographical areas within one local authority in Scotland.  Standard maternity care |
| Feldman, 2013  (39) | To investigate the health impact of dispersal and relocation on pregnant women and new mothers seeking asylum.  Face-to-face structured interviews with women experiences from women who had been dispersed in pregnancy. | 20 women, most had been dispersed or relocated during a pregnancy in the previous three years. 2 women whose dispersal was stopped on medical grounds, and 1 woman who was not dispersed but was being kept in Initial Accommodation. UK.  Standard maternity care |
| Goodwin, 2018  (40) | To explore midwife–woman relationships for migrant and minority ethnic women in the UK.  A focused ethnography including semi- structured interviews with, fieldwork in the local migrant Pakistani community and local maternity services, observations of antenatal appointments, and reviews of relevant media. | 9 migrant Pakistani women  Standard maternity care |
| Hatherall, 2016  (41) | To explore the factors which influence the timing of the initiation of a package of publicly-funded antenatal care for pregnant women living in a diverse urban setting  qualitative study involving individual interviews focus groups | 32 women from four community groups (Bangladeshi, Somali, Lithuanian and Polish).  Standard maternity care |
| HESTIA, 2018  (42) | To shine a light on the most serious and overlooked aspects of modern slavery in London today.  Mixed methods including in-depth interviews | 10 women who were victims of modern slavery and gave birth whilst their case was considered in the National referral mechanism  Standard maternity care |
| Jomeen, 2013  (43) | To explore Black and minority ethnic (BME) women’s experiences of contemporary maternity care in England.  A secondary analysis of open-ended questionnaire responses from a UK wide survey. | 368 women who self-identified as BME responded with open text.  Standard maternity care |
| Lephard, 2016  (44) | To explore the maternity care experiences of local, pregnant, asylum-seeking women, to inform service development.  Phenomenological approach using semi-structured interviews | 6 women seeking asylum who had used UK maternity services in the preceding year.  Four of the six women were from sub-Saharan Africa and two were from Eastern Europe.  Standard maternity care |
| Malouf, 2017  (45) | To explore the lived experiences of pregnancy, childbirth, prenatal and postnatal care and services received by women with learning disabilities in the UK, including their expressed information and support needs relating to maternity care.  In-depth semi structured interviews | 9 women with learning disabilities who were pregnant or had given birth within the last 3 years in the UK  Standard maternity care |
| McLeish, 2018  (46) | To explore the maternity care experiences of mothers with multiple disadvantages | 40 mothers with multiple disadvantage  Standard maternity care |
| Montgomery, 2015  (47) | To inform practice by exploring the impact that childhood sexual abuse has on the maternity care experiences of adult women.  Narrative study from a feminist perspective using in-depth interviews. | 9 women who were sexually abused in childhood  Standard maternity care |
| Moxey, 2016  (48) | To explore how Somali women exposed to female genital mutilation experience and perceive antenatal and intrapartum care in England.  A descriptive, exploratory qualitative study using face-to-face semi structured interviews. | 10 Somali women residents in Birmingham, who had accessed antenatal care services in England within the past 5 years.  Standard maternity care |
| Phillips 2015  (49) | To explore and gain insight onto the expectations and experiences of women with a pre-existing diagnosis of mental illness, of their first booking appointment.  Semi-structured interviews | 12 participants with mental illness  Standard maternity care |
| Phillimore 2016  (50) | To explore the reasons new migrant women, book late to maternity care and do not attend antenatal follow-up appointments.  Questionnaire with qualitative and quantitate response and in-depth interviews. | 82 questionnaires were completed by recent migrant women. 13 new migrant women were interviewed.  Standard maternity care |
| Puthussery, 2010  (51) | To explore the maternity care experiences and expectations of United Kingdom (UK)-born ethnic minority women.  Qualitative in-depth interviews | 34 UK-born mothers of Black Caribbean, Black African, Indian, Pakistani, Bangladeshi and Irish descent  Standard maternity care |
| Thomson, 2013  (52) | To offer a critical discussion from a public health perspective of service user’s experiences of antenatal care services.  A qualitative, descriptive study using group and individual semi-structured interviews | 92 participants with ‘social vulnerabilities’  Standard maternity care |
