# Supplementary material for: How do women with social risk factors experience United Kingdom maternity care? A realist synthesis
Source: Birth. 2019 Aug 5;46(3):461–74. doi: 10.1111/birt.12446 (PMC6771833; doi:10.1111/birt.12446)
Supplement: Supplementary file 2 [file BIRT-46-461-s002.docx]

| **Programme Theories** | **Middle Range Theory** |
| --- | --- |
| **RESOURCES**  **ACCESS AND SYSTEM BARRIERS**  If women receive written information (in their preferred language) about how to access maternity services and what their care will offer and are able to do this directly rather than through a GP, then barriers around NHS administration and/or postal delays will be overcome and antenatal care will commence earlier in pregnancy. (1), (3), (4), (7), (9), (10), (11), (12), (14), (16), (18), (19), (20), (22)  If women are able to register with maternity services and GP’s without extensive documentation or evidence of a permanent address, then they could access care earlier in pregnancy, reduce stress and fear of disclosure to agencies or individuals who might put them at risk. This will, in turn, improve early access to abortion services. (4), (7), (9), (11), (12), (14), (16), (20)  If maternity care incorporated early pregnancy care (from conception/confirmation of pregnancy), then women would not view it as a package of care for viable and continuing pregnancies and therefore see value of accessing care early in pregnancy to seek support and advice regardless of whether or not they intend to continue the pregnancy. (7), (10), (11), (12)  If women registering late for maternity care are fast-tracked through the system to ensure an early antenatal appointment and time to build a relationship with a known midwife, then the potential impact of her late booking on birth outcomes can be minimised. (7), (20)  **INTERPRETATION SERVICES**  If HCP’s listen to women’s choices about interpreter services, for example a female, an anonymous, or a trusted interpreter, then barriers to their use and effectiveness will be reduced and women would feel more comfortable discussing sensitive subjects and disclosing concerns with their healthcare provider, improving safety. (1),(5),(7), (9), (12), (13), (14), (18), (20), (21)  If women have easy, immediate telephone access to interpreter services to register with services, arrange or reschedule appointments, organise travel to appointments, and access to properly translated materials, then inequity in information received and a key communication barrier will be overcome, and women will be better able to access services. (11), (14), (18), (20), (21)  **EDUCATION**  If antenatal education was culturally sensitive including information that is relevant to women’s individual needs at an appropriate gestation, (for example child friendly settings and classes without the presence of men) and provide an opportunity to meet a small team of midwives providing their care, then more women would engage with the classes and be better informed about their birth choices. (1),(2), (6), (8), (15), (19)  If basic, evidence-based information about maintaining a healthy pregnancy, and procedures/routines is readily available, easy to understand, and translated into new migrant languages, then women would be better informed, able to provide consent, and have less reliance on the internet and advice from friends and family. (6), (8), (15), (20)  If women have more face-to-face time with a health professional to discuss their lifestyle, then they will better understand the impact of risky behaviours, as many do not engage with or understand information provided in leaflets. (22), (13)  **PRACTICAL SUPPORT**  If HCP’s support women in difficult circumstances to address the emotional and practical challenges they face by providing them with new skills, knowledge and resources (for example help to resolve infant feeding challenges, provision of breast pumps, bottles and storage bags, reassurance, and motivation to abstain from illegal substances), then they will be better prepared to overcome challenges and internalise this as evidence of care and concern that HCP’s feel towards them. (2), (12)  If midwives have the time, resources and skills to coordinate and facilitate practical support to meet women’s wider needs (this may include providing information about statutory procedures, contacting social workers, writing letters on their behalf, as well as coordinating, attending and facilitating meetings with other statutory agencies (e.g. Social care, Housing departments, Home Office)), then women will be better informed of unfamiliar processes and better equipped and supported in difficult circumstances. (2), (11),(14), (20)  If HCP’s are educated in maternity benefits available for socially vulnerable women, and able to provide advice around practical matters such as housing, employment, education and care of other children and family members, then women would see more value or purpose in accessing services earlier in pregnancy and further financial hardship and distress for the women could be avoided. (9), (10), (14), (16), (20)  **CONTINUITY**  If women can access a known midwife 24/7 via a phone call or text message, then they will be better able to engage with services, care will be more personalised, they will feel more cared for, and are less likely to have to repeat their history and experience a variation of responses/advice. (2),(3), (4), (7), (14), (15), (19), (21), (22)  If women feel they have a continued supportive presence throughout pregnancy and the perinatal period, either with a midwife, GP or other healthcare professional, then they will feel better supported and have reduced feelings of anxiety, increased sense of control, and enhanced self-beliefs and wellbeing. (2), (4), (6), (7), (10), (13), (14), (15), (16), (17), (19), (22)  If women are offered continuity of care and are able to build a trusting relationship with their midwife, then underlying social risk factors can be explored and care individualised to their needs to improve engagement and empowerment so that women are better able to express or restate their expressed wishes and concerns. (7), (13), (17), (16)  **COMMUNITY/LOCATION**  If HCP’s work in a small geographical area where they are visible and become known by other members of the community, religious networks and other ‘gatekeepers’, then they can work together to develop trust, facilitate family and community-centred care, and educate the community with evidence-based information and dispel common, harmful myths. (3), (14), (18), (20), (21), (22)  If HCP’s are familiar with local charities, food banks, befriending programmes and support services then they will be able to introduce women to these services in order to provide the most supportive networks possible before they are discharged from maternity care and women will be better able to integrate into the community. (2), (9), (12), (14), (20), (21)  If a programme provides physical and social opportunities for women to receive flexible, needs-led care, where the time and place of appointments is co-planned (for example at home, community or a hospital setting), then women will have the best chance to access timely antenatal care, feel listened to and empowered by taking control of their care. (2),(3),(7),(8), (9), (14), (15), (20)  If services are flexible for women who live socially complex lives and move location frequently, or for those who have no access to a telephone or resources to travel far away to a hospital, for example local drop in services, appointments at home, or at the weekend, not at school times for single mothers, not during working hours for women working illegally, then their engagement with services can be improved as much as possible (1), (2), (4), (9), (12), (20), (22).  If midwives are able to visit women at home in the antenatal period, then they will not only overcome barriers such as women unable to travel to appointments, but also be able to assess the living conditions of women to provide more individualised, holistic care. (9), (14), (15), (20)  **TIME / ACTIVE PARTICIPATION**  If antenatal care provides reassurance through clinical checks, effective preparation for labour, an opportunity for socialising with other mothers, and women are encouraged and given the time and resources required to ask questions about their pregnancy and care, then women will see the service as beneficial, feel like active participants and engage with their healthcare providers. (2), (8), (10), (13), (15), (16), (17), (20), (22)  If healthcare professionals give information in an unbiased way, and listen to women’s choices, questions, and decisions, then women will be able to make informed choices about their pregnancy, feel a sense of control and being listened to, and demonstrate their ability to make appropriate choices. (10), (15), (16), (20)  If women accessing busy maternity services with rushed staff feel that they are being ‘processed through a system by professionals who follow procedures without really noticing the woman in front of them’, then they will not feel cared for, supported, or valued and have a perceived lack of social support. (3), (13), (16), (15)  If models of care were flexible, appropriately staffed, and midwives had full autonomy over their working days and appointments, then women would not perceive the pressure of time and feel more able to disclose information and midwives would have improved attitudes as they would not be working to unrealistic time constraints. (3), (4), (13), (16), (20), (21)  **MULTIDISCIPLINARY WORKING/COMMUNICATION**  If there are clear paths of communication across different trusts and services such as GP, gynaecology, maternity services, social care and mental health services is seamless, then women would be able to access care earlier in pregnancy and experience less fragmentation and disassociation between the services. (7), (9), (10), (11), (19), (22)  If models of care facilitated the development of effective support networks for women throughout their pregnancy through working with family members and multidisciplinary support services (social workers, health visitors, support workers, children’s centres and voluntary sector agencies), then that established support network will enable new mothers to flourish and become confident and successful parents. (9), (15), (16) | **Resources**  Access/System barriers  Education  Flexibility  Community  Continuity of care  Multi-disciplinary working  Support  Staffing  Time |
| **AGENCY/ RECIPRIOCITY**  If a trusting relationship develops through open discussion and story sharing between women and their HCP, then women will have confidence in their HCP, trust their advice, and benefit from their support. (2), (3), (4), (13), (16)  If a programme offers advocacy, midwife attendance at meetings, and other forms of emotional support during interactions with social care then women will feel supported and informed of unfamiliar processes (2), (6), (10)  If healthcare professionals inform women of their right to choice, through education and providing the evidence-based information women need to exercise that choice, then they will be empowered, and their self-confidence increased through shared decision making and would not feel as through accessing care equates to relinquishing control through perceptions of manipulation and coercion by the healthcare professional. (4), (6), (10), (11), (13),(15)(16), (17)  If maternity care encompasses the foundations of woman-centred care: working with women as partners, respecting their expertise of their own body, needs and baby, and making decisions based upon individuals rather than stereotypes or entrenched professional norms, then women will be more situated in a context of control rather than disempowerment. For some women this may also avoid disempowerment, feelings of being pressurised, ignored and excluded, long lasting psychological trauma, and increase bonding between a mother and her baby. (4), (6), (8), (11), (13), (16), (22)  If healthcare professionals recognise that socially deprived women are more likely to experience paternalistic maternity care, as passive recipients, then the HCP can personalise care and strive to involve women in planning and decision making to ensure women are active, respected participants. This can in turn improve the self-confidence these women often lack in situations where there is a power imbalance. (8), (13), (15), (16), (17)  If women are encouraged by healthcare professionals to raise concerns in an easy and confidential manner and escalate those concerns if they are not satisfied with the response, then they will not only feel empowered and listened to, but potential adverse outcomes could be avoided. (7), (15)  If women feel they are under surveillance, or that asking questions/disclosing information will cause their healthcare provider to judge them, then they will perceive their care to be stressful and disempowering, rather than a supportive, informative preparation to parenthood and will feel that it is safer not to ask for help. (13), (15), (16) | **Agency/ Power**  Advocacy  Surveillance  Participation  Knowledge  Control  Intergenerational vulnerability  Empowerment  Social Capital |
| **TRUST**  If women receive more personal continuity in their care, then they will develop feelings of trust and confidence in their healthcare professionals and have more meaningful interactions (for example disclosing sensitive information or exploring the context of women’s requests/concerns). (3), (7),(8), (9), (10), (11), (13), (16), (17), (19), (21), (22)  If women perceive their support network to be invested in their ability to parent successfully, and receive practical, tailored advice and positive affirmations, then they will feel less scrutinised and feel better able to seek support and advice when needed. This in turn will demonstrate how they are able to seek appropriate help and parenting advice. (2), (6), (15), (22)  If women have a level of trust and confidence in their HCP’s and do not fear judgement, for example their concerns are listened to on an individual level, they receive meaningful information, and they are able to rebook missed appointments with ease and without reproach, then they will perceive the maternity environment as a place of safety and their engagement with flexible services will improve. (13), (15), (16), (19), (20), (21) (22)  CHARACTERISTICS OF THE HCP  If women have the opportunity to get to know their healthcare professional and perceive them to be respectful, understanding, kind, and helpful, then women will feel cared about and cared for, empowered and better able to express or restate their expressed wishes and concerns. (5), (7), (8), (13), (21)  Conversely, if women with low socio-economic status experience paternalistic care through being denied choice and perceive HCP’s as lacking warmth, patronising, arrogant, and stigmatising, then they will remain disempowered, feel undervalued and their low self-confidence will increase. (15)(16)  If HCP’s recognise pregnancy as a time of emotional fragility and added stress for women living socially complex lives and can empathize and respectfully respond to their individual needs, then more may emerge from their maternity experience feeling empowered rather than violated.(5), (17),(21) | **Symbolic Interactionism**  Relationship  Self-disclosure  Value  Trust  Empathy  Holism  Respect  Individualised  Assets/Strengths  Intergroup contact theory |
| CANDIDACY  If women who have had a previous traumatic experience and/or have lost confidence in the system and approach services tentatively are able to develop a trusting relationship with their HCP, then their trust in the system may be restored and their engagement with services improved. (4), (5), (6), (7), (9), (11), (12), (17),  If midwives acknowledged that some women, especially those with unintended pregnancies, are undecided whether they want to continue with the pregnancy when they access services, then they would be better prepared to support and advise women in an objective manner and women would not view maternity services as exclusive to those with continuing pregnancies (7), (11), (15)  If the value of accessing maternity services for the purpose of monitoring, prevention and support is communicated across the communities in which women live, then women would not view the purpose if the service as simply the treatment of ill health and access care earlier in pregnancy. (10), (11), (20)  If midwives and women are able to get to know each other and build a trusting relationship, then the midwife will be more aware of a woman’s social situation and able to provide individualised, holistic support without labelling women or making assumptions about their needs based on a perceived cultural background. (5), (6), (10), (14), (15)  If midwives acknowledge the importance of culture and the influence of family members on women’s experience of pregnancy, then they will be able to personalise care around the needs and cultural norms of the family unit and avoid potential conflicts in offering advice that does not reflect a cultural norm (1), (5), (10), (13), (18), (21)  If HCP’s work within a community where they are immersed in local cultures different to their own, or the hospital environment, then they will become culturally sensitive, women will not feel their cultural needs are being disregarded in favour of the western medical model and inequities in access, engagement, the uptake of screening, and antenatal education will be reduced. (1), (10), (13), (18), (21)  If women with low socioeconomic status experience discriminatory, or impersonal care, then their often already fragile self-confidence can be further undermined, making them feel they are not good enough to parent. (6), (15),(16) | **Discrimination**  Intersectionality  Candidacy  Familiarity  Conflicting cultures  Stigmatisation  Labelling  Assumptions  Social isolation  Disconnectedness  Cultural competence |

**Programme theories specific to social risk factor**

| Asylum seekers /Refugees/ Migrants (1), (2), (5), (9), (10), (11), (12), (14), (16), (18), (20), (22) | If newly arrived immigrant women or those with refugee status have access to language concordant group educational programmes or befriending services, they will be better informed, empowered and less isolated. (3), (5), (14)  If maternity services and immigration services (UKBA) had a standardised method of communication during pregnancy, then women who are detained or dispersed could be followed up and appropriate care plans made or handed over to other trusts, and the UKBA better able to ask midwives whether these women were safe to travel or had other health needs. (9), (14), (20), (21)  If pregnant women seeking asylum have access to information about healthcare services that are often unfamiliar to them, whilst in initial accommodation, such as a direct phone number to specialist midwifery services, then the value of care will be better understood, access and engagement improved, and costly health emergencies avoided. (9), (14), (20)  If women who are seeking asylum are not dispersed during pregnancy or the postnatal period, then their physical and mental health would not suffer as a result of being isolated from a known community that includes partners, friends, support for giving birth, childcare, churches, temples and mosques, networks of children established at school, GPs and midwives. (9), (12), (14), (20)  If women with immigration problems who are worried that they can be tracked by immigration authorities and their babies removed if they registered with maternity services (believing their presence in the UK would be evident if their name was entered into an electronic database) are protected by a firewall established between maternity and immigration services so that it is widely understood, as in Portugal, that maternity professionals will not report undocumented migrants or failed asylum seekers, then access, engagement and overall safety of the mother and her unborn will improved. (20)  If asylum seeking women were able to suspend their asylum claim until six weeks after childbirth, instead of after 12 weeks of pregnancy, then attendance and engagement with one NHS trust would be facilitated and potential adverse outcomes associated with lack on engagement avoided. . (20)  If maternity services recognise that migrants without recourse to public funds often work lengthy hours at below minimum wage and lack the protection of employment laws which at least in theory means that migrants who are “on the books” are entitled to attend appointments, then services could be adapted to meet their needs. This might include drop in clinics, weekend and evening appointments, local services, telephone access. (20) |
| --- | --- |
| BME (1), (3), (5), (7), (10), (11), (12), (13), (14), (16) (18), (20), (21), (22) | If women possess a cultural sense of docility, then they may accept care and information without questioning it., in addition to this if women are language discordant, then they may be concerned about being perceived as a ‘problem-patient’ for their healthcare provider and appear ‘docile’ and ‘compliant’ (5)  If importance is placed only on the culture behind a health issue, then healthcare providers might inadvertently hand the problem over to the patient as a private matter. (5)  If women believe that pregnancy outcomes are controlled by external factors, such as a supernatural power and lack education to make informed choices, then their sense of personal autonomy and control may be undermined.(5)  If ethnic minority women born in the UK have an absence of language barriers, and are familiar with the NHS system, then they do not want or feel the need to receive specialist care based on their ethnicity. (21) |
| Child protection (2), (6), (15), (16), (22) | If women and family members are given the information and opportunity to make choices around their pregnancy, for example place of birth, pain relief, mode of delivery, discharge from hospital, requesting changes in healthcare professional, then they will feel more empowered and in control of their pregnancy. For those whose parenting capacity is being assessed, this can help them to demonstrate how they process information and make choices based on what is best for them and their baby. (15), (16), (19)  If midwives explain the reasoning behind safeguarding concerns and the process of assessment, then women with social service involvement will be better prepared and supported for the often stressful and intense process of child protection assessment. (6), (15)  If women are looked after by a known healthcare professional then they are less likely to receive conflicting advice from numerous healthcare professionals. This may have direct consequences on child protection outcomes for women whose parenting is being assessed by social care. (15)  If women who are undergoing parenting assessments by social care are looked after in pregnancy by a known midwife they trust, then they will be more open to disclose information about their physical and emotional wellbeing, and this honest dialog may support their parenting assessment through demonstration of help-seeking, learning and making positive decisions. (15)  If women who are undergoing parenting assessments are looked after by a known midwife whom they trust, and feel is invested in their chance of successfully parenting their child, then they will have more confidence in their own abilities and perceive less discrimination. (15)  If women are not informed about social care processes and parenting assessments, and lack information needed about pregnancy, birth and parenting, then they will be unclear about what standards of behaviour they are being judged against. This can leave women feeling frustrated, disempowered and marginalised, and potentially lose parental responsibility of their children. (15) |
| Childhood sexual abuse (17) | If healthcare professionals listen to unspoken messages (for example in requests for female staff or planned caesarean sections), recognise distress and validate women’s experiences, then open communication can be enhanced, and women will feel their healthcare professional has a genuine interest on them as an individual. (17) |
| Disabled (6), (22) | If women with a disability and/or experiencing abuse feel that a referral to social care could be beneficial to them and their needs as a new mother, then they will be more likely to disclose, and their fear of judgement lessened. (6)  If each pregnancy is viewed as an individual, unique, journey, rather than labelled as low risk/high-risk/normal/abnormal, then care for women with a disability would not be dominated by the social norms of a traditional medical model, rather than those of a holistic, woman-centred care. (6) |
| Domestic abuse (2), (6), (16), (22) | If women with a disability and/or experiencing abuse feel that a referral to social care could be beneficial to them and their needs as a new mother, then they will be more likely to disclose, and their fear of judgement lessened. (6)  If women experiencing domestic abuse have a trusting relationship with their healthcare provider, then they will be more likely to understand statutory reporting and gain benefits through additional support and safety mechanisms. (22) |
| FGM (18) |  |
| Homelessness (2), (9), (12), (21) |  |
| Learning difficulties (6), (15), (22) | If healthcare professionals caring for women with learning disabilities had prior knowledge of different forms of sharing complex information, then women would receive individualised information and advice, and would not perceive care to be judgemental (6), (15) |
| Mental health (2), (16), (19), (22) | If midwives are well informed about the mental health issues experienced by many women with complex social histories and have clear processes in place to refer women to effective perinatal support services, then women will be able to access these support services early in pregnancy and prevent these mental health issues worsening in the postnatal period. This will better prepare women for motherhood and improve the mother-infant bonding process. (9), (16), (19) |
| Non-English speaking (1), (11), (12), (14), (18), (20), (22) | If women who have survived rape, sexual assault or other trauma have access to an interpreter they trust and feel comfortable with, for antenatal, postnatal appointments, and labour care, then they will be better informed, more able to communicate concerns and flashbacks or other psychological responses during labour (9)  If women do not trust discussing personal matters with an interpreter, despite whether the interpreter was a stranger or someone from within their own social community, then language barriers will continue (5)  If interpreter services were available on request and for emergency appointments, and a lack of need not assumed, then women would be better able to communicate effectively with their healthcare provider. (13) |
| Single parents (16), (22) | If services are flexible for women who live socially complex lives and move location frequently, then their engagement with services can be improved as much as possible, for example appointments at home, or at the weekend, not at school pick up/drop off times for single mothers, not during working hours for women working illegally. (20) |
| Social deprivation (3), (7), (8), (9), (12), (14), (16), (22) | If a programme provides direct and practical support and material items for women as they began parenthood, such as breast pumps and infant feeding supplies, phone ‘top ups’, clothes, baby equipment, nappies, money for travel as well as toys for older children, then they will be enabled to meet their and their babies’ material needs. (2)  If women who have few resources, such as no phone credit, have direct, easy access to a midwife through a free phone number, or free technology such as WhatsApp, skype, etc, then their anxieties will be allayed and engagement with services improved. (7), (14), (22)  If midwives were able to conduct appointments in the woman’s home or local GP or community centre, then women who are living in poverty with little resource for public transport would be better able to engage with maternity services. (9), (14), (20), (22)  If women with low socio-economic status experience paternalistic care through being denied choice, then they will remain disempowered, feel undervalued and their low self-confidence will increase.(16), (17) |
| Social isolation (2), (14), (16), (22) | If intrapartum care cannot be provided by a known midwife, then midwives should be able to refer women who are alone to subsidised doula services for support and advocacy during labour. (14) |
| Substance misuse (2), (22) |  |
| Teenager (7), (16), (22) |  |
| Trafficked women/ Modern slavery (4), (12), (16) | If HCP’s understand trafficked people’s rights and entitlements, then as well as encouraging greater use of maternity services, this knowledge may also promote earlier use of maternity, and other healthcare services. (4)  If access to maternity services is controlled and observed by traffickers, then women may access maternity services late in pregnancy and be unable to disclose for fear of punishment by their traffickers. (4)  If trafficked women do not know how to access NHS services, or they fear of being charged a fee as a non UK resident, then they will have poorer access and engagement with services(4)  If women are offered ongoing, targeted support for their mental and physical health and parenting of infants conceived in the traumatic setting of trafficking, then inter-generational transmission of vulnerability, and adverse mental and physical child health outcomes can be prevented. (4)  If pregnant victims of modern slavery were able to access perinatal mental health services at the earliest gestation possible, then they will be better able to overcome barriers to recovery and better prepared for the psychological impact of pregnancy, childbirth and motherhood. (12)  If women who are being exploited have open, direct access to maternity services and emergency support, then their desire to protect their unborn child may give them the courage to escape to a safe point of contact. (12)  If women with a history of trauma and abuse are able to develop a trusting relationship with their healthcare provider, then barriers to accessing and engaging with health services will be overcome through individualised care plans and sensitivity around intimate examinations. (12), (17) |
| Travelling community (22) |  |

1. ALSHAWISH, E., MARSDEN, J., YEOWELL, G. and WIBBERLEY, C., 2013. Investigating access to and use of maternity health-care services in the UK by Palestinian women. British Journal of Midwifery, 21(8), pp. 571-577.
2. BALAAM, M. and THOMSON, G., 2018. Building capacity and wellbeing in vulnerable/marginalised mothers: A qualitative study.
3. BEAKE, S., ACOSTA, L., COOKE, P. and MCCOURT, C., 2013. Caseload midwifery in a multi-ethnic community: the women's experiences. Midwifery, 29(8), pp. 996-1002.
4. BICK, D., HOWARD, L.M., ORAM, S. and ZIMMERMAN, C., 2017. Maternity care for trafficked women: Survivor experiences and clinicians’ perspectives in the United Kingdom’s National Health Service. PLoS one, 12(11), pp. e0187856.
5. BINDER, P., BORNÉ, Y., JOHNSDOTTER, S. and ESSÉN, B., 2012. Shared language is essential: communication in a multi-ethnic obstetric care setting. Journal of health communication, 17(10), pp. 1171-1186.
6. BRADBURY-JONES, C., BRECKENRIDGE, J.P., DEVANEY, J., KROLL, T., LAZENBATT, A. and TAYLOR, J., 2015. Disabled women’s experiences of accessing and utilising maternity services when they are affected by domestic abuse: a critical incident technique study. BMC pregnancy and childbirth, 15(1), pp. 181.
7. CALLAGHAN, M., BULLER, A.M. and MURRAY, S.F., 2011. Understanding ‘late bookers’ and their social circumstances. British Journal of Midwifery, 19(1), pp. 7-13.
8. DOCHERTY, A., BUGGE, C. and WATTERSON, A., 2012. Engagement: an indicator of difference in the perceptions of antenatal care for pregnant women from diverse socioeconomic backgrounds. Health Expectations, 15(2), pp. 126-138.
9. GOODWIN, L., HUNTER, B. and JONES, A., 2018. The midwife–woman relationship in a South Wales community: Experiences of midwives and migrant Pakistani women in early pregnancy. Health Expectations, 21(1), pp. 347-357.
10. Feldman, R., 2013. When maternity doesn't matter: dispersing pregnant women seeking asylum. *Reproductive Health Matters*, *21*(42), pp.212-217.
11. HATHERALL, B., MORRIS, J., JAMAL, F., SWEENEY, L., WIGGINS, M., KAUR, I., RENTON, A. and HARDEN, A., 2016. Timing of the initiation of antenatal care: an exploratory qualitative study of women and service providers in East London. Midwifery, 36, pp. 1-7.
12. HESTIA, 2018. Underground Lives: Pregnancy and Modern Slavery. HESTIA. London.
13. JOMEEN, J. and REDSHAW, M., 2013. Ethnic minority women's experience of maternity services in England. Ethnicity & health, 18(3), pp. 280-296.
14. LEPHARD, E. and HAITH-COOPER, M., 2016. Pregnant and seeking asylum: Exploring women's experiences ‘from booking to baby’. British Journal of Midwifery, 24(2), pp. 130-136.
15. MALOUF, R., MCLEISH, J., RYAN, S., GRAY, R. and REDSHAW, M., 2017. 'We both just wanted to be normal parents': a qualitative study of the experience of maternity care for women with learning disability. BMJ open, 7(3), pp. e015526-2016-015526.
16. MCLEISH, J, and REDSHAW, M., 2018. Maternity experiences of mothers with multiple disadvantages in England: A qualitative study. *Women and Birth*.
17. MONTGOMERY, E., POPE, C. and ROGERS, J., 2015. A feminist narrative study of the maternity care experiences of women who were sexually abused in childhood. Midwifery, 31(1), pp. 54-60.
18. MOXEY, J.M. and JONES, L.L., 2016. A qualitative study exploring how Somali women exposed to female genital mutilation experience and perceive antenatal and intrapartum care in England. BMJ open, 6(1), pp. e009846-2015-009846.
19. PHILLIMORE, J., 2016. Migrant maternity in an era of superdiversity: new migrants' access to, and experience of, antenatal care in the West Midlands, UK. Social science & medicine, 148, pp. 152-159.
20. PHILLIPS, L. and THOMAS, D., 2015. The first antenatal appointment: an exploratory study of the experiences of women with a diagnosis of mental illness. Midwifery, 31(8), pp. 756-764.
21. PUTHUSSERY, S., TWAMLEY, K., MACFARLANE, A., HARDING, S. and BARON, M., 2010. ‘You need that loving tender care’: maternity care experiences and expectations of ethnic minority women born in the United Kingdom. Journal of health services research & policy, 15(3), pp. 156-162.
22. THOMSON, G., DYKES, F., SINGH, G., CAWLEY, L. and DEY, P., 2013. A public health perspective of women's experiences of antenatal care: an exploration of insights from a community consultation. Midwifery, 29(3), pp. 211-216.
